# Beam intensity and stability control on a modified clinical linear accelerator for FLASH irradiation

**DOI:** 10.1088/1361-6560/adf8ac

**Published:** 2025-08-14

**Authors:** Yuewen Tan, Naresh T Deoli, Andrew D Harken, David J Brenner, Guy Garty

**Affiliations:** 1 Radiological Research Accelerator Facility, Columbia University, 136 S. Broadway, Irvington, NY 10533, United States of America; 2 Center for Radiological Research, Columbia University, New York, NY 10032, United States of America

**Keywords:** FLASH-RT, clinical linac, modified linac, ultrahigh dose rate, electron irradiation, UHDR beam stability, UHDR repeatability

## Abstract

*Objective.* The FLASH effect has gained significant attention in radiobiology and radiation oncology due to its potential to improve therapeutic outcomes by delivering ultra-high dose-rate (UHDR) irradiations. Understanding UHDR biological mechanisms can also contribute to the development of biodosimetry and radiological medical countermeasures. However, achieving stable and reproducible high-current UHDR electron beams has been reported to be challenging with modified clinical linear accelerator (Linac) systems, and has not been systematically studied. *Approach.* We investigated how key standing-wave linear accelerator parameters, including electron gun current, pulse-forming network voltage, and auto-frequency control, affect the stability of electron beam intensity on a modified Varian Clinac 2100 C. We also developed a parameter-tuning method to adjust beam intensity and improve beam stability. *Main results.* This approach enabled (1) fine-tuning of dose-per-pulse without modifying the physical setup and (2) reduction of beam fluctuations, particularly during cold starts. These improvements enhanced both pulse-by-pulse stability and trial-by-trial reproducibility. The resulting stability was validated through multiple biological experiments. *Significance.* This work offers practical guidance for improving UHDR beam stability and reproducibility, as well as enabling intensity tuning in modified clinical linear accelerators. It can support the development of more reliable preclinical FLASH irradiators, thereby contributing to the advancement of FLASH research.

## 1. Introduction

Since the first *in vivo* demonstration of the FLASH effect in 2014, related studies have quickly become a major interest in radiobiology and radiation oncology (Favaudon *et al* 2014, Catherine Vozenin *et al* 2019). Understanding the mechanisms of FLASH is critically important to the future design of FLASH radiotherapy and its optimal clinical use, for example, to investigate the scope and robustness of the FLASH effect and the possibility of fractionation (Loo *et al* 2024). In recent years, great effort has been put into revealing the mechanisms of the FLASH effect (Montay-Gruel *et al* 2017, Limoli and Vozenin 2023) and applying it in preclinical and clinical therapeutic studies (Montay-Gruel *et al* 2018). Beyond applications in FLASH radiotherapy, understanding the effects of ultrahigh dose-rate (UHDR) radiation is critical in the field of radiation protection and biodosimetry. In a nuclear detonation, a large fraction of the dose will be delivered in under a microsecond (Garty *et al* 2017). In order to validate biodosimetry assays and, importantly, test medical countermeasures, under realistic exposure conditions, an irradiation platform that can reliably deliver a predetermined dose in a single microsecond-scale pulse is required.

Since 2017, numerous clinical electron linear accelerators (Linacs) have been modified to perform UHDR irradiation and repurposed for FLASH studies (Schüler *et al* 2017, Lempart *et al* 2019, Rahman *et al* 2021, Garty *et al* 2022, Xie *et al* 2022, Dal Bello *et al* 2023, Oh *et al* 2024, Zhou *et al* 2024). Additionally, specially designed electron Linacs have been developed for FLASH research (Faillace *et al* 2021, Liu *et al* 2023). Several review articles summarize the current status, challenges, and technical considerations in adapting clinical Linacs for FLASH applications (Farr *et al* 2022, Romano *et al* 2022, Schüler *et al* 2022, Joshua No *et al* 2023, Subiel *et al* 2024). At the Radiological Research Accelerator Facility (RARAF), a decommissioned Clinac 2100 C (Varian Medical Systems, Palo Alto, CA, USA) was received from a donation in 2019, and subsequently modified into an electron irradiator for FLASH studies as well as biodosimetry high dose-rate studies, which require a system that can reliably deliver a predetermined dose within a single pulse, mimicking exposure from an improvised nuclear device (Garty *et al* 2022). The modified Clinac has been operated in service mode, using 9 and 16 MeV electron mode, and the 6 and 15 MV photon modes without the x-ray target. Over the past few years, this system has been employed in various *in vitro* (Garty *et al* 2022, 2023, Shuryak *et al* 2023) and *in vivo* studies (Padilla *et al* 2024, Pannkuk *et al* 2024).

Our previous monitoring of beam intensity over a one-year period in 2022 revealed systematic variations correlated with the modulator’s operational status. These fluctuations were influenced by both the beam-on time and the environmental conditions of the accelerator hall, particularly when initiating a cold start. This variability introduced significant trial-by-trial uncertainty, making it difficult to establish reproducible experimental conditions, particularly in experiments requiring identical dose-per-pulse (DPP), pulse count, and dose distribution. During a continuous beam run, the dose rate variations at low DPP caused by inconsistency of the beam intensity can be mitigated through adaptive pulse repetition rate adjustments. However, in UHDR beam configurations, where doses are delivered in a single microsecond-scale pulse or a few pulses (up to 40 pulses for a clinically relevant dose using our system), nonuniform DPP within a pulse train or across trials might compromise the dosimetric consistency and biological response reproducibility. Additionally, for each beam run, the RF and klystron are first activated, and the output beam is triggered once the RF has stabilized; after the output pulse train, the RF and klystron are shut down. Restarting the beam too soon can disturb the dynamics of the pulse-forming network and acceleration cavities, potentially affecting the initial beam conditions and contributing to pulse-by-pulse dose instability. These challenges align with previous reports highlighting reproducibility and stability concerns in modified Linac systems (Jaccard *et al* 2017, Lempart *et al* 2019, Ashraf *et al* 2022, Garty *et al* 2022, Gonçalves Jorge *et al* 2025), which restricted our ability to perform experiments that demand high repeatability and larger UHDR irradiation volumes.

Moreover, in high pulse current mode, the built-in ionization chamber would be saturated, which hindered our previous attempts to optimize the beam intensity in high-current mode (namely the SuperFLASH mode in our previous report (Garty *et al* 2022)). A high beam current monitor is necessary to integrate for better beam operation (Gonçalves Jorge *et al* 2022, Liu *et al* 2023).

In this study, we systematically investigated key accelerator parameters, including the gun current (GUNI), pulse-forming network voltage (PFNV), and auto-frequency control (AFC), to optimize the UHDR beam performance. We propose here a parameter-tuning-based approach to fine-tune the beam intensity and stabilize the variation, particularly during cold starts. This method not only improves trial-by-trial reproducibility but also enables active control of beam intensity. Maximizing the beam intensity allows biological experiments that require larger UHDR volumes.

## 2. Methods

### 2.1. Clinac setup, dosimetry, and beam monitor

In this section, we provide a summary of our retired Varian Clinac 2100 C modifications, high-current-mode electron beam operation (photon mode with the x-ray target removed from the beam path), and dosimetry, in addition to our previous descriptions (Garty *et al* 2022).

#### 2.1.1. Mechanical setup and modifications

**Figure 1.**
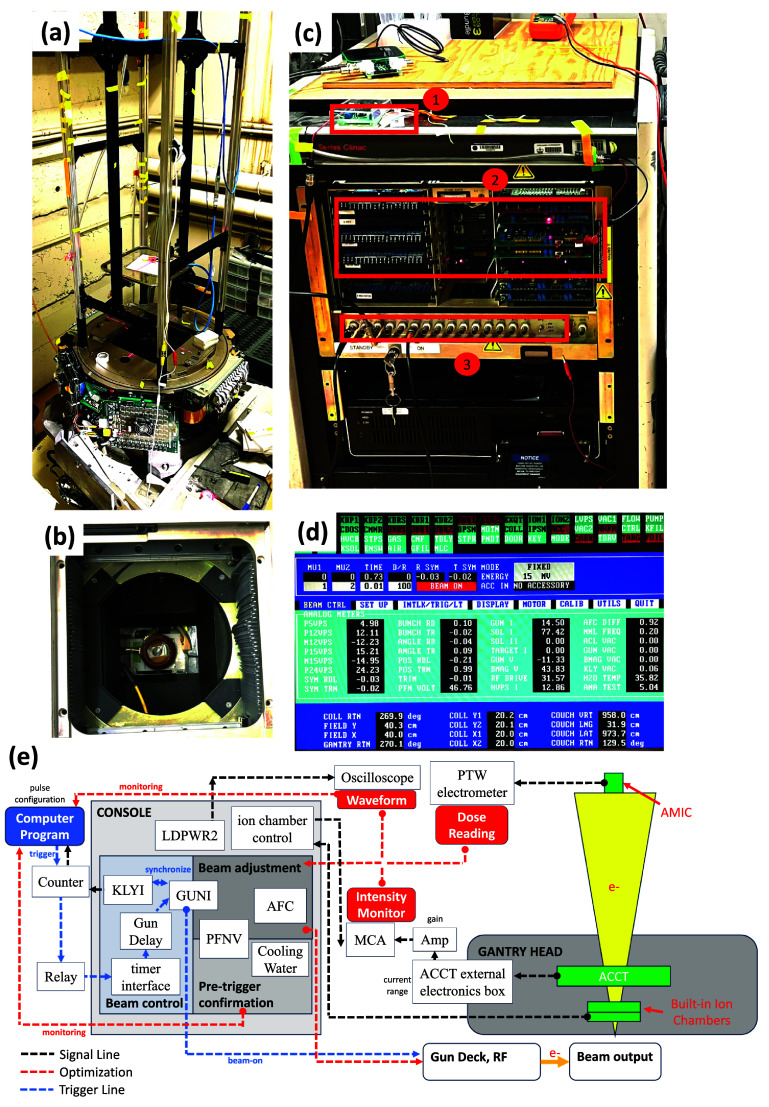
(a) The optical rail experiment platform was mounted on the gantry head, oriented at 180^∘^, where the beam points up vertically. The crosshair sample holder can move along the rail to achieve a specific SSD, with a laser crosshair used for precise sample alignment. (b) The ACCT detector is installed inside the gantry head, positioned on top of an aluminum holder directly above the built-in ionization chamber. (c) Console at the accelerator control room. The beam triggering module, PCB card deck, and analog signal panel are highlighted in the picture and labeled 1, 2, and 3, respectively. (d) Screen in service mode. Analog signals are displayed for parameter adjustment and beam operation. This specific screenshot displays the optimal parameters for 15 MV mode. (e) Schematic diagram of essential components for beam control, monitoring, and optimization.

The gantry head was fixed in place with the beam direction pointing vertically up (figure 1(a)). The patient couch was removed. The scattering foil was removed from the carousel, and the x-ray target control was overridden such that the target is positioned outside the beam field. To maximize the initial beam intensity, the accelerated electron beam was scattered only by the exit window and the built-in ionization chambers before entering the air. A high-bandwidth in-air current transformer (ACCT, manufactured by Bergoz Instrumentation, Saint-Genis-Pouilly, France) was installed at the beam exit inside the gantry head (figure 1(b)) to monitor the real-time beam current. The detector range was set to 100 mA with a rise time of approximately 120 ns (10%–90%).

#### 2.1.2. Beam operation

A computer and controlling PCB cards are enclosed in a console cabinet, shown in figure 1(c). Electronic signals can be accessed at the console, and several PCBs were adjusted or modified with an add-on circuit board. The machine was operated in service mode using the Varian Clinac operating software. Unnecessary interlocks were disabled to allow beam customization while preserving essential protections against accelerator damage. Details of the overridden interlocks are summarized in the supplemental section. Real-time analog readings from the machine are presented on the console computer’s screen for parameter adjustment, monitoring, and beam optimizations, shown in figure 1(d).

**Figure 2.**
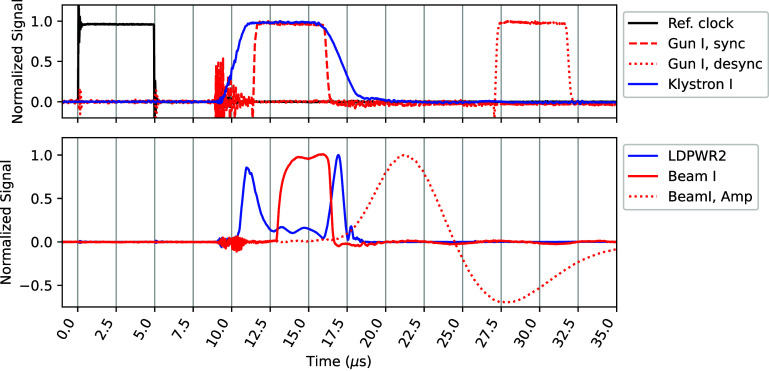
The signals were measured using a digital oscilloscope at the analog signal panel of the console. The signals were triggered and aligned by the 180 Hz SYNC signal (top, black) as a reference. Desynchronized (top, red dotted) and synchronized (top, red dashed) GUNI were compared with the KLYI (top, blue). When the GUNI and KLYI overlap, the RF power accelerates the electrons. The beam current waveforms of 15 MV mode (bottom, red solid) were collected from the ACCT detector, together with its corresponding LDPWR2 waveform (bottom, blue). The beam current signals were integrated by an amplifier of shaping time 3 *µ*s, and the heights of amplified signals (bottom, red dotted) were collected using a multi-channel analyzer as a measure of beam pulse energy. The three dips of the LDPWR2 waveform characterized the energy dissipation in the accelerator cavities due to the choice of RF frequency, and determined the shape of the beam output, which were further optimized.

The beam pulses were controlled by enabling or disabling the GUNI delay at the timer interface PCB using a relay (figure 1(c-1)). When the electron gun and Klystron pulses (GUNI and KLYI, respectively) were synchronized, an output electron beam radiation was generated; when there was a delay between the two, no radiation was generated. This ensured the control of the beam on/off without perturbing the dynamics of RF in acceleration cavities and allowed a more reproducible action of the beam output. These signals can be accessed at the console unit of the Clinac. We demonstrate the relevant beam control signals in figure 2. Additional details about beam pulse control and triggering are provided in the supplemental section. The repetition rate of the KLYI is 180 Hz, and thus the maximum mean dose rate is given by, $\overline{\dot{D}} = $$\overline{\text{DPP}}\cdot 180\,\text{Hz} \cdot n/(n-1)$, where $\overline{\text{DPP}}$ is the mean DPP, and *n* ($\unicode{x2A7E}2$) is the number of pulses.

#### 2.1.3. Beam monitors

In low-current mode (the electron modes of the Clinac), the built-in ionization chambers monitored the beam pulse intensity reliably. One built-in ionization chamber output at the Console (test Point TP1 on the ion chamber control card in figure 1(c-2)) was connected to a multi-channel analyzer (MCA) to record pulse height as the beam pulse intensity. For high-current modes (the photon modes, without a target), the amplified ACCT signal replaced the built-in ionization chamber signal in the MCA for beam intensity measurements. A digital oscilloscope (Digilent, Pullman, Washington, United States) was used to collect the ACCT’s waveform signal, while an amplifier (Advanced Measurement Technology, Oak Ridge, Tennessee, United States) with 3 *µ*s shaping time and adjustable gains amplified and integrated the signal, and sent it to the MCA.

#### 2.1.4. Dosimetry

The dosimetry was conducted using Gafchromic EBT3 and EBTXD films (Ashland Specialty Chemicals, Wayne, New Jersey, United States). Films were calibrated with nominal 16 MeV electron beams and an Advanced Markus ionization chamber (AMIC) from PTW (Freiburg, Germany) at a low dose per pulse (DPP). The DPP for calibration is typically 10–20 mGy/pulse, but can be higher if necessary. The AMIC, operating at −300 V polarization, was calibrated to the absorbed dose in water using a ^60^Co source at the MD Anderson Accredited Dosimetry Calibration Laboratory every two years. The AMIC ion recombination effects became significant above approximately 0.1 Gy/pulse, which were previously investigated (Petersson *et al* 2017, Garty *et al* 2022, Liu *et al* 2024). These studies demonstrated that the AMIC can be used reliably beyond low DPP regimes when properly corrected. In our practice, we typically use the chamber at DPP levels up to 0.5 Gy/pulse. All film setups and scanning measurements were optimized based on our previously reported methodology and some other publications (Borca *et al* 2013, Jaccard *et al* 2017, Niroomand-Rad *et al* 2020, Liu *et al* 2023)

### 2.2. Beam intensity control

For a standing-wave electron linear accelerator, the electron gun current GUNI and corresponding pulse-forming network voltage PFNV greatly affect the output beam intensity and stability. Gauging the dynamics of the RF power via monitoring the cavities’ power usage (load power 2, LDPWR2) can also be used to optimize the beam pulse shape and pulse-by-pulse stability. In this work, we surveyed the parameters required to obtain a controllable electron beam intensity in the 16 MeV mode (the original low-current electron mode) and the 15 MV mode (originally a high-current photon mode, modified to deliver electrons by removing the target).

#### 2.2.1. GUNI and PFNV

The RF pulse power modulation is controlled by the precise timing of thyratron discharge (deQing), which releases PFN stored energy to Klystron. The PFNV parameter, adjusted via the ‘programmable PFNV’ potentiometer at the energy-mode program PCB card (figure 1(c)-2), directly regulates the deQing time and beam pulse energy. The PFNV value was obtained from the analog signal display on the console screen. It is shown with two decimal places and fluctuates in increments of 0.04, due to variations in the high-voltage charging source of the PFN. Similarly, the GUNI parameter via the ‘programmable GUNI’ potentiometer at the energy-mode control card can control the electron gun pulse current. The GUNI fluctuates much less and appears to exhibit only a gradual decrease. Each energy mode has its predefined programmable ranges for GUNI and PFNV, on the energy-mode control PCB card, allowing for optimal matching of GUNI with the corresponding PFNV. We span the PFNV and GUNI range for the 16 MeV mode and 15 MV energy mode, and measured their mapping beam intensity.

#### 2.2.2. AFC

**Figure 3.**
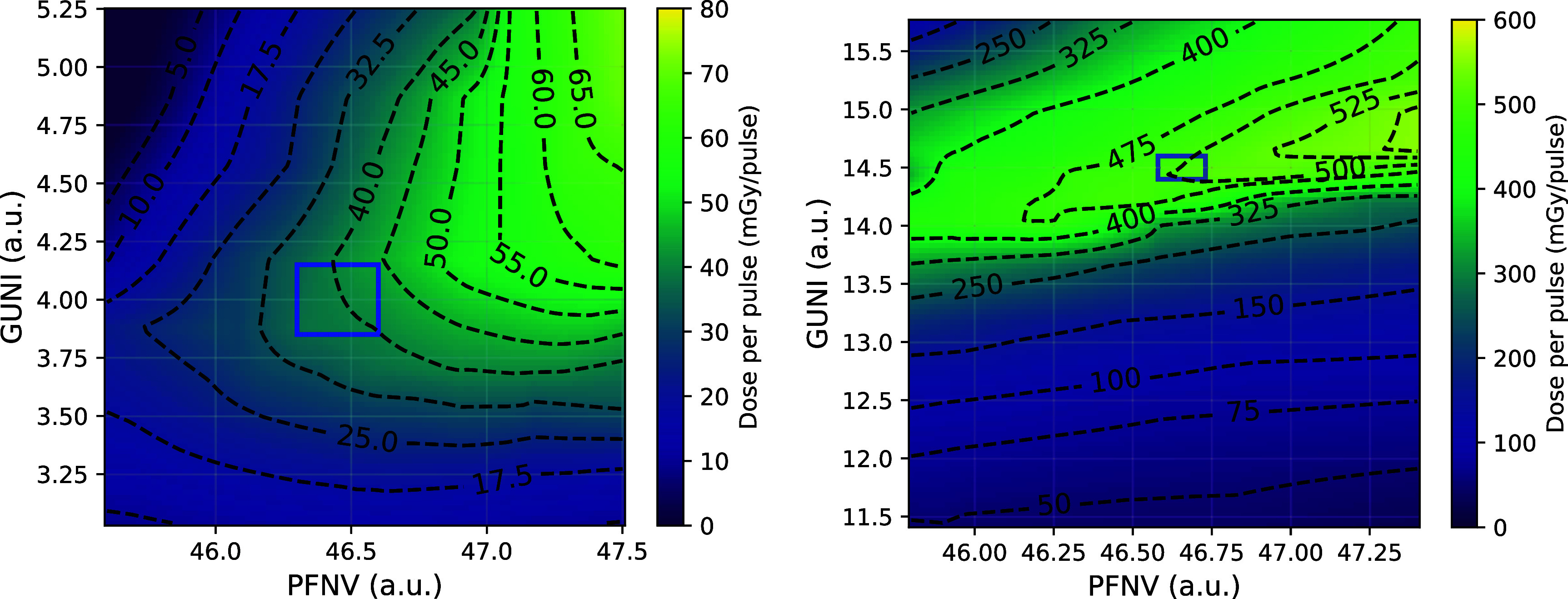
DPP control map as a function of PFNV and GUNI with isodose contours and optimal operational window. The measurements were taken at an SSD of 170 cm using the 16 MeV (left) and 15 MV (right) modes. For DPP values less than 200 mGy, where the ion pair recombination has no significant effect, ACCT and AMIC were used for measurements and verification, while for higher DPP values, Gafchromic films and ACCT were employed. Parameters in the optimal operational windows produce a high-intensity beam while being practically highly reproducible.

In a standing-wave Linac, while PFNV stabilizes, RF power and scattered electrons induce energy into the acceleration cavity, altering local thermal conditions of the microwave cavities and causing shifts in their resonance frequencies. The AFC system compensates for these shifts, improving transmission efficiency during beam acceleration. The AFC module adjusts the RF frequency based on feedback between AFC1 and AFC2 terminals, which monitor resonance frequency discrepancies between the front and rear RF cavities. The RF system’s load condition is tracked by the LDPWR2 terminal (at figure 1(c-3)). This signal accounts for RF’s forward and reflective power and indicates the cavity’s performance. Proper programmable AFC settings ensure LDPWR2 waveform remains in optimal shape (Sloop *et al* 2024), indicating efficient beam pulse acceleration at a certain RF frequency. Each energy mode has its own optimal programmable AFC parameter.

#### 2.2.3. Pulse width control

The beam intensity is also determined by the electron gun pulse width. For this study, we fixed the nominal electron gun pulse width to 4.5 *µ*s at the electron gun deck. The output beam pulse width can be further fine-tuned at the logical interface PCB (figure 1(c-2)) by aligning the Klystron current (KLYI) and GUNI pulse (see supplemental figure S1). If a larger GUNI pulse width is used, the GUNI delay must be adjusted to optimize KLYI-GUNI pulse alignment.

#### 2.2.4. Beam-on conditions

A schematic block diagram in figure 1(e) demonstrates an outline of optimization (red dashed lines) and beam triggering (blue dashed lines) components. The black dashed lines represent general signal transmission paths for reference. The system is coordinated through a computer program interfaced with the Clinac console. The beam optimization process is done by actively monitoring the MCA, AMIC, and LDPWR2 signals to improve or adjust the beam intensity by iteratively tuning the GUNI, PFNV, and AFC parameters. The cooling water temperature, PFNV value, and LDPWR2 waveform are monitored to ensure full reset and stabilization during the pre-trigger phase. The beam is triggered by the computer program, and the signal will follow the trigger line through the relay, then passed to the console, and eventually lead to the beam output.

To ensure consistent beam output, we follow these specific steps for each irradiation: (1) resetting the cavity temperature to base operating temperature by waiting $\thicksim$3 min while monitoring the cooling water temperature, (2) activating the RF with unsynchronized GUNI and KLYI, while monitoring the PFNV and LDPWR2, (3) triggering the beam (synchronize GUNI and KLYI) after PFNV is stabilized, and (4) making sure LDPWR2 is at a higher electron beam transmission efficiency state just before triggering the beam. In figure 2(bottom) we present an LDPWR2 waveform alongside its corresponding beam pulse current waveform for a high current 15 MV beam, collected by the ACCT detector.

### 2.3. Irradaitions

#### 2.3.1. Characterization measurements

For the maximum DPP vs SSD measurements, we used the 15 MV mode and selected GUNI-PFNV parameters that maximized the beam intensity. Stacked solid water slabs (or water equivalent polystyrene RW3, LAP laser, Boynton Beach, FL, USA) of 5 cm total thickness were placed at a specific SSD, and the DPP was measured at a depth of 1 cm in the solid water slabs using Gafchromic film. Dose distribution measurements were performed using Gafchromic films in a setup similar to the maximum DPP vs SSD measurements.

#### 2.3.2. Beam intensities in biological experiments

Beam intensities were measured using either Clinac’s built-in ionization chamber or the ACCT, recorded, and analyzed across all 93 biological experiments conducted from May 2022 to March 2025 using the 9 MeV, 16 MeV, or 15 MV modes. UHDR irradiations were performed in the 9 MeV and 15 MV modes. These experiments include irradiations of clonogenic assays, comet assays, patient-developed organoids, skin tissues, hindbrain of mice, gastrointestinal of mice, upper-body mice, lower-body mice, and total-body mice (Shuryak *et al* 2023, Padilla *et al* 2024, Pannkuk *et al* 2024). Additionally, we performed UHDR total-body mouse irradiations in combination with neutron irradiation. In total, over 1950 irradiations were performed using various pulse numbers, SSDs, and shielding conditions.

## 3. Results

### 3.1. Beam characterization

#### 3.1.1. GUNI-PFNV heat map and DPP control

The DPP as a function of PFNV and GUNI at SSD of 170 cm was measured using 16 MeV and 15 MV modes, as shown in figure 3. The heat map was generated through linear interpolation between measured values. Compared to GUNI, the PFNV value exhibits more variation and primarily governs the beam intensity variability. The base fluctuation of PFNV was found to be 0.04. To minimize beam intensity variation, the PFNV should be set in a low-gradient region in the heat map. Higher PFNV values inherently fluctuate more than lower PFNV values due to the instability of the Klystron charging current. In 15 MV mode, when the PFNV value exceeds a certain threshold (which depends on the cold start conditions), it tends to drop and oscillate within ±0.3, which significantly impacts the consistency of the output beam. A more effective strategy for stabilizing the beam is to maintain the PFNV below the threshold level. This issue will be further explained in the section on the record of beam intensity records for biological experiments.

**Figure 4.**
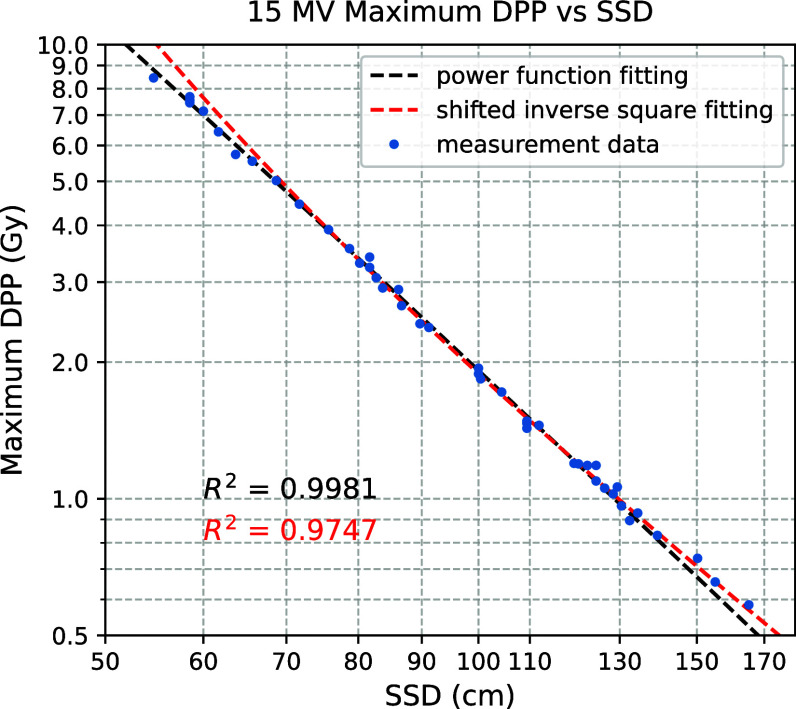
Maximum DPP at various SSDs. Both axes were plotted on log scale. Each data points were measured by a Gafchromic film inserted between water equivalent polyethylene slabs at depth of 1 cm and irradiated by the 15 MV beam (GUNI = 14.50 and PFNV = 46.79, triggered within the optimal triggering window). The measurement data points were either fitted by the shifted power function or inverse square function of the SSD. The fitting function is $\text{DPP} = 45410\cdot(SSD-4)^{-2.66}$ for the power function, and $\text{DPP} = 1190\cdot (SSD-20.5)^{-2}$ for the shifted inverse square function, where DPP and SSD are in units of Gy and cm, respectively.

Figure 4 illustrates the maximum DPP versus SSD. The DPP vs SSD does not follow the exact inverse square law due to air scattering and beam energy heterogeneity. A shifted inverse square fit can account for the region where SSD is greater than 60 cm. For a smaller SSD, the power function fit is more reliable.

Using the plot, DPP can be adjusted by moving the SSD. This method can be useful for experiments requiring a small field or those where minor dose distribution changes are negligible. However, for experiments requiring consistent DPP and dose distribution, the SSD must remain the same, and adjusting the combination of GUNI and PFNV can change the DPP. For the convenience of FLASH irradiation setup, four SSDs (35, 60, 100, and 170 cm) were selected as fixed locations depending on the maximum required DPP.

#### 3.1.2. Dose distribution at fixed SSDs

**Figure 5.**
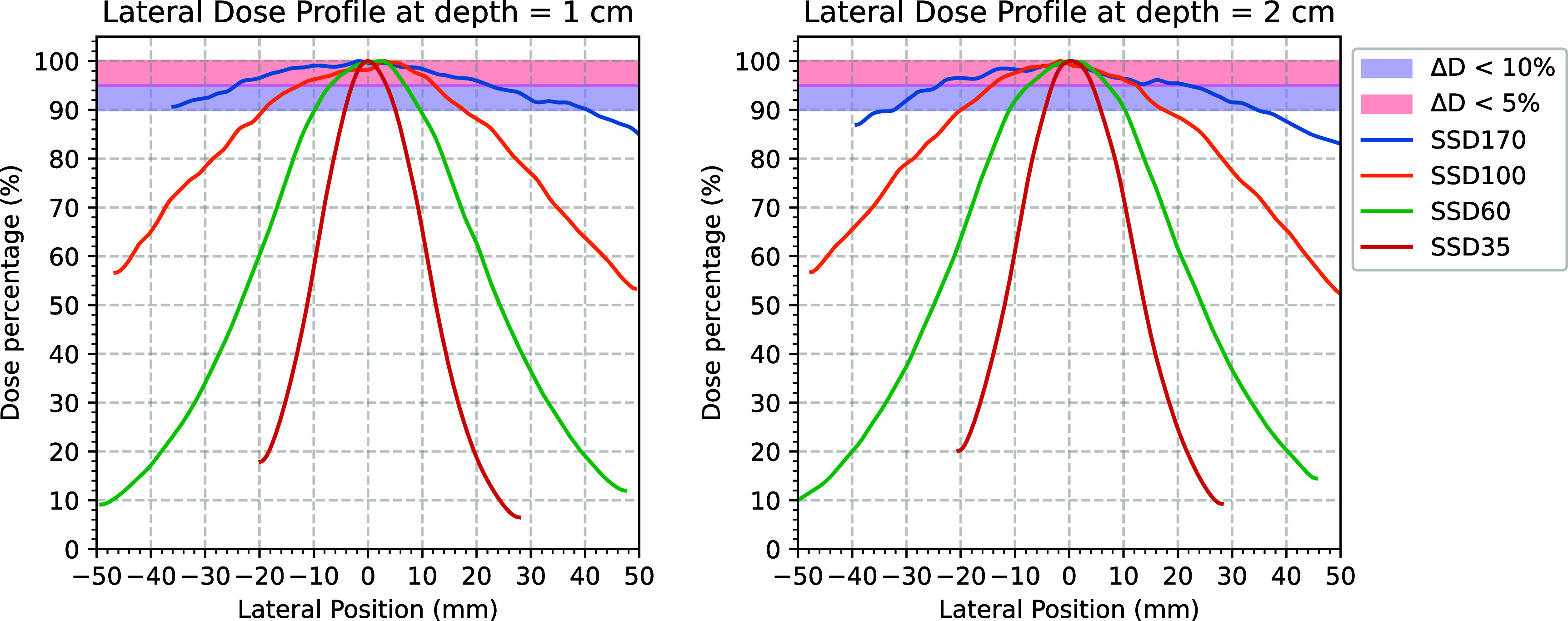
Lateral dose profiles at SSDs of 35, 60, 100, and 170 cm and depths of 1 and 2 cm of phantom in 15 MV mode. The blue and red regions indicate the dose region above 90% and 95% maximum, respectively.

**Figure 6.**
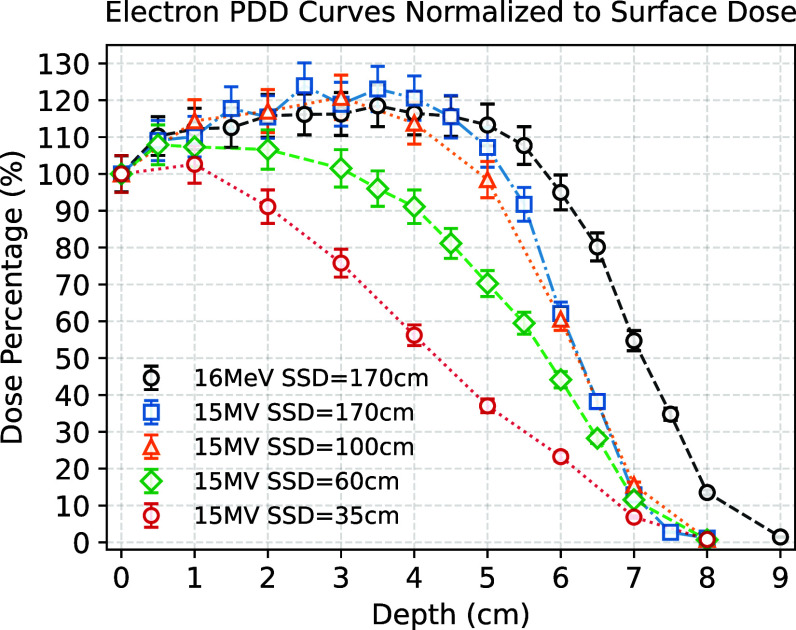
Axial PDD at SSDs of 35, 60, 100, and 170 cm using 15 MV mode, and SSD of 170 cm using 16 MeV mode. Each data point was read from the averaged film dose in a 5 mm circular region, centered at the axial location of each film. The error bars were the combination of 5% uncertainties of film reading and dose variance in the target region.

Figure 5 plots the lateral dose profiles at depths of 1 cm and 2 cm for four SSDs. Figure 6 plots their axial percentage-depth-doses (PDDs). These figures serve as reference points for setting up experiments at the designated SSD stages. The standard beam quality parameters for these two energy modes are summarized in table 1.

**Table 1.** Quality of 15 MV and 16 MeV electron beam.

Energy mode	SSD (cm)	R_90_ (cm)	R_50_ (cm)	FW90M (cm)	DPP (Gy)	$\dot{\mathrm{D}}$ (Gy s^−1^)
15 MV	170	4.0	6.0	8.0	0.5	90
	100	3.5	6.0	3.7	2.0	360
	60	3.2	5.6	2.0	7.0	1260
	35	1.5	4.3	0.9	20	3600

16 MeV	170	5.0	6.9	8.5	0.05	—

#### 3.1.3. Role of AFC in DPP

**Figure 7.**
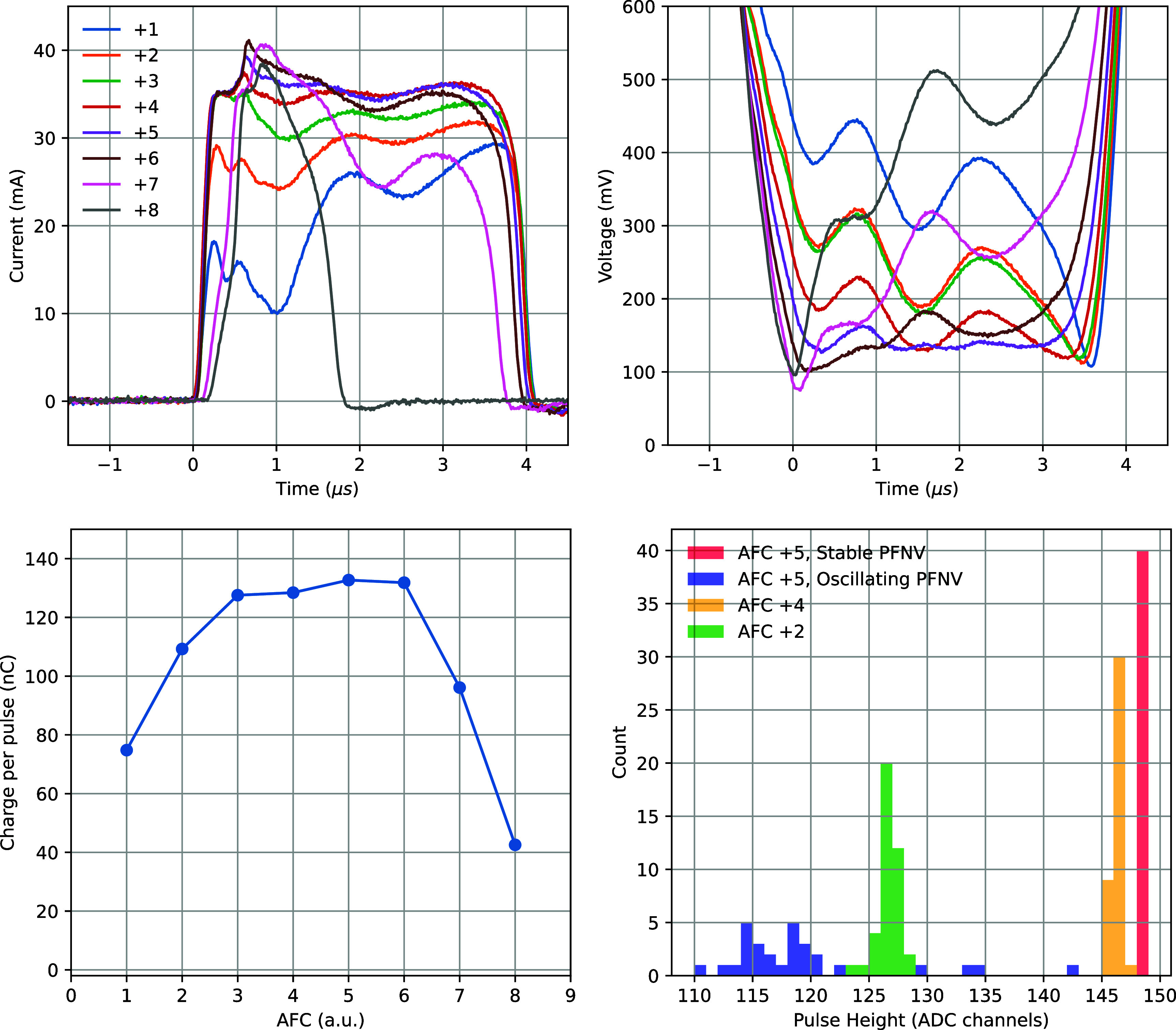
Top: beam currents and corresponding LDPWR2 waveforms using various AFC values from +1 to +8. These values are in arbitrary units based on the programmable AFC potentiometer increments. Bottom left: total charge of a beam pulse using various AFC values. Bottom right: pulse height distributions of 40 pulses of different beam settings.

The tuning of AFC directly alters both the LDPWR2 waveform and the resulting beam current waveform (as well as the DPP). In figure 7(top), the beam current and LDPWR2 waveforms are compared for eight different AFC potentiometer settings (with GUNI = 14.79 and PFNV = 46.72). The corresponding total charge per pulse is plotted in figure 7(bottom left). The AFC values are in arbitrary units based on the programmable AFC potentiometer increments. In this case, the optimal AFC range is between +4 and +6. Adjusting the AFC parameter shifts the RF frequency, thereby controlling the transmission of each beam pulse. A larger RF offset will cause the beam current distribution to become skewed. The top two plots demonstrate this gradual change and highlight the optimal LDPWR2 waveform for achieving the best beam current waveform. Monitoring the LDPWR2 can help ensure that the beam passes through the optimal RF frequency.

### 3.2. Beam stability and intensity enhancement

#### 3.2.1. Pulse-by-pulse variability

The stability of PFNV primarily determines the DPP variation within a pulse train. When PFNV remains stable, the AFC setting may still introduce minor spreading in the DPP distribution. As shown in figure 7 bottom right, even with AFC configured within its optimal range, significant DPP variation is observed across the 40 pulses if PFNV is unstable. A slight offset in the AFC setting can subtly reshape the beam current waveform, leading to increased DPP variation. The greater the AFC offset, the larger the spread in the DPP distribution.

#### 3.2.2. Trial-by-trial variability

**Figure 8.**
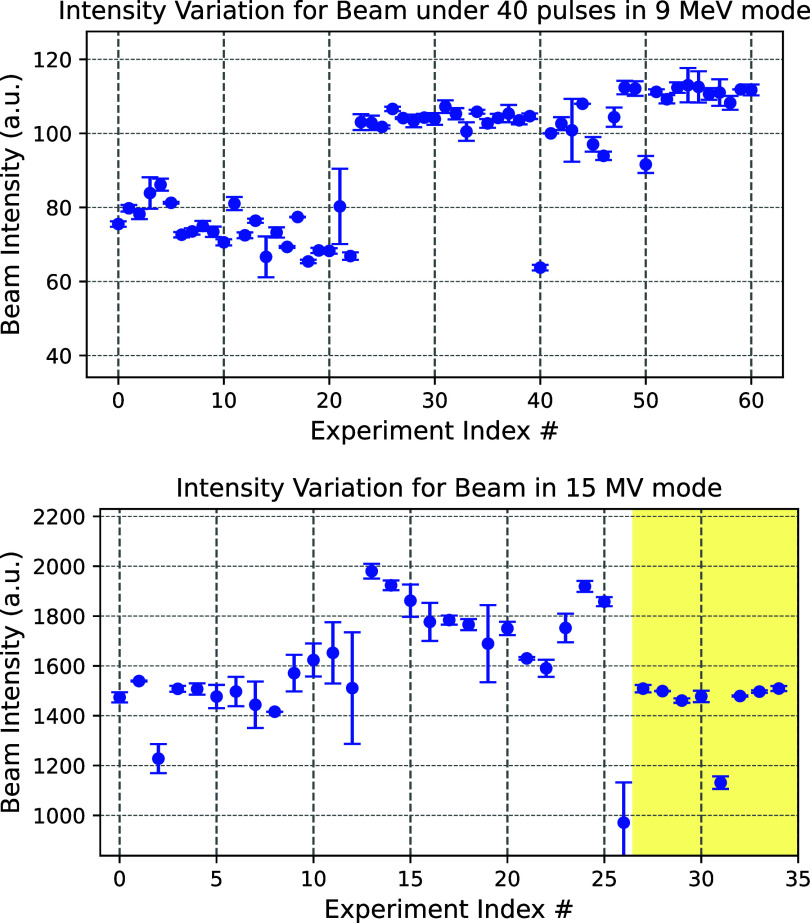
Overall mean beam intensity variation for each biological experiment using 9 MeV (top) and 15 MV modes (bottom). The plots primarily focus on the beam intensities of UHDR irradiation (under 40 pulses). The horizontal axis indicates the index of the UHDR experiment in time order. Before March 2024, all the UHDR irradiations were performed by the 9 MeV beam; after that, the 15 MV beam was used. The highlighted area is where we started to implement the most recent operational procedure for 15 MV mode.

The recorded beam intensities of all experiments are summarized in figure 8. This study examines beam stability in the context of UHDR irradiation, particularly for experiments with fewer than 40 pulses. A complete beam intensity data set of all irradiations can be found in the supplemental figure S2, including large pulse count irradiations. In figure 8 top, the first 22 experiments conducted in 9 MeV mode were unadjusted and previously reported (Garty *et al* 2022). From the 23rd experiment onward, we systematically gauged the PFNV parameter to enhance pulse beam stability, and in later experiments, we slightly increased the GUNI to generate higher beam intensity. This adjustment significantly reduced trial-by-trial instability during beam operations. The outliers in the 9 MeV plot were due to maintenance issues, such as the thyratron degrading and dying out over a couple of experiments, rather than beam operational errors. Excluding those data points, the trial-wise variance of the beam intensity (a.u.)^3^3Referring to the MCA channel number recorded per centivolt. This value is proportional to the actual beam intensity under fixed ADC and amplifier settings but was reported in arbitrary units, as it did not correspond to a directly measured physical quantity. improved from $ 74.6\pm5.6$ to $ 104.1\pm1.7$. Increasing GUNI provided further improvement of beam intensity to $111.4\pm1.4$ while maintaining consistency.

Building on our success in stabilizing the low-current 9 MeV mode, we applied a similar approach to the high-current 15 MV mode. Initially, beam intensity variance was high due to an elevated PFNV, which caused instability when the KLYI high-voltage power supply struggled to sustain current during beam-on stages. A high GUNI setting further amplified PFNV fluctuations ±0.3, due to its high beam intensity gradient in PFNV direction. This effect was reflected in our 15 MV’s 13th to 25th experiments (figure 8 bottom), in which the beam intensities were consistent on each experiment day but changed after another cold start. To mitigate this, we adopted a new strategy-lowering both GUNI and PFNV parameter values to achieve a more stable beam (GUNI = 14.50; PFNV = 46.72). The maximum DPP was reduced by approximately 20% on average compared to the largest possible DPP. To date, this revised approach has been implemented in the eight most recent 15 MV experiments, yielding excellent beam stability. In the 31st experiment, which required 80% of the original beam intensity, we successfully reduced the beam intensity and maintained stability using the GUNI-PFNV beam intensity map shown in figure 3(right). This methodology effectively ensures consistent FLASH beam production, allowing us to conduct previously challenging UHDR experiments.

## 4. Discussion

In this study, we demonstrated that beam intensity and stability are primarily governed by the proper selection of GUNI and PFNV, and can be further optimized by adjusting the AFC based on the LDPWR2 signal. While previous studies have reported non-uniformity of DPP within pulse trains and between trials, without dedicated beam control, their solution primarily focused on AFC tuning alone (Ashraf *et al* 2022, Sloop *et al* 2024, Zhou *et al* 2024, Gonçalves Jorge *et al* 2025). Our work provided a more comprehensive analysis of how GUNI, PFNV, and AFC collectively influence beam intensity. This approach is not only useful for the Varian Clinac 2100 C, but also for other clinical Linacs using standing-wave design. This method is particularly effective in high-current modes, where instability is more pronounced. To our knowledge, this is the first study to investigate beam intensity variation across three years of trials, supported by detailed experimental records and a complete history of beam conditions.

Variations in internal accelerator parameters can occur each time the machine is started from standby mode (a cold start), likely due to environmental influences. These fluctuations can impact beam stability and must be accounted for in experimental setups. At the start of operation, the GUNI parameter is initialized at its maximum programmed value from the energy mode program PCB. Over 3–4 h, GUNI gradually drifts down by 2% of the initial value, leading to a decrease in beam intensity. For example, the initial GUNI value at 14.50 could decrease to 14.30 over 4 h, which would reduce the beam intensity by nearly 10% when using the optimal PFNV. Waiting for GUNI’s stabilization would be a necessary warm-up procedure for long-duration experiments, ensuring consistent irradiation conditions.

Another source of variation following a cold start is the drift of PFNV values. The PFNV values can shift by up to ±0.2, affecting the resulting DPP when adjusting PFNV and GUNI. To mitigate this issue, it is recommended to check the lowest programmable PFNV value of the energy mode and align it with the reference lowest PFNV value in figure 3. In some instances, the PFNV can constantly oscillate between two values, resulting in highly unpredictable and heterogeneous output beam pulses, as we presented in figure 7(bottom right). This indicates the instability of Klystron high-voltage power, and can typically be resolved by rebooting the system, ramping up and conditioning the high-voltage power supply with a low-current energy mode (e.g. 6 MeV or 9 MeV) before switching to higher energies.

There are other operational procedures that can alter the beam intensity or increase its instability. For example, seasonal temperature fluctuations in the accelerator hall, particularly in winter, have been observed to cause SF_6_ gas leakage in our system. A drop in SF_6_ pressure leads to a reduction in beam intensity, even when operating within available pressure limits. Regular monitoring and maintenance of SF_6_ levels are necessary for preventing such losses.

The uncertainty of the beam intensity mostly comes from the fluctuation of the PFNV value. At the beginning of an irradiation, the Klystron will be started first until the PFNV reading is stabilized. Once the PFNV is stabilized at its optimal value, the PFNV value may still fluctuate by approximately ±0.04, leading to an overall beam intensity uncertainty of 1%–2%.

To enhance beam stability and reduce uncertainties, systematic conditioning procedures have been implemented before each irradiation session. This includes performing pre-experiment PFNV calibration, executing controlled warm-up cycles for GUNI stabilization, monitoring AFC-controlled LDPWR2, and monitoring SF_6_ levels in real-time.

To further examine beam stability and pulse shape in greater detail, the procedure can be integrated with real-time beam diagnostics. By implementing signals from the ACCT, the system can provide immediate feedback on beam fluctuations, enabling dynamic adjustments to improve dose accuracy and guarantee the trial-by-trial repeatability. Future developments may include automated feedback systems capable of adjusting PFNV in real time, ensuring consistent beam intensity throughout prolonged irradiation sessions.

Building on our newly developed technique for active DPP control, we are now able to conduct a wider range of UHDR experiments that were previously limited. Our default choice of SSD for UHDR irradiations is 170 cm, and most of the experimental setups were characterized at this position. Dose distributions have been characterized for both 48- and 96-well plates, achieving DPP values at the center up to 0.7 Gy/pulse (over 120 Gy s^−1^). This setup is also compatible with a hypoxia incubator chamber (STEMCELL Technologies, Cambridge, MA), allowing for gas environment control. This setup is convenient for a variety of *in vitro* studies that are using well plates. The UHDR field size at SSD of 170 cm is sufficient to cover the full body length of a mouse and can also be used with shielding for partial-body irradiation. For juvenile mice, the field can accommodate up to three animals simultaneously, significantly improving experimental throughput and time consistency. Active control of DPP now enables single-pulse studies and investigations involving variable DPP. The flexible control of DPP also helps UHDR instrumentation development, allowing for testing and calibration of emerging UHDR detectors, such as semiconductor and luminescent detectors designed for high DPP applications.

## 5. Conclusion

In this study, we optimized UHDR electron beam performance through systematic adjustments of GUNI, PFNV, and AFC parameters, resulting in flexible DPP control and significantly improved beam stability for FLASH experiments. This advancement provides a practical parameter-tuning method for preclinical FLASH irradiators converted from medical linacs, and supports consistent UHDR biological studies, contributing to the development of FLASH radiotherapy toward clinical translation.

## Data Availability

All data that support the findings of this study are included within the article (and any supplementary information files).
